# Antibody escape of SARS-CoV-2 Omicron BA.4 and BA.5 from vaccine and BA.1 serum

**DOI:** 10.1016/j.cell.2022.06.005

**Published:** 2022-07-07

**Authors:** Aekkachai Tuekprakhon, Rungtiwa Nutalai, Aiste Dijokaite-Guraliuc, Daming Zhou, Helen M. Ginn, Muneeswaran Selvaraj, Chang Liu, Alexander J. Mentzer, Piyada Supasa, Helen M.E. Duyvesteyn, Raksha Das, Donal Skelly, Thomas G. Ritter, Ali Amini, Sagida Bibi, Sandra Adele, Sile Ann Johnson, Bede Constantinides, Hermione Webster, Nigel Temperton, Paul Klenerman, Eleanor Barnes, Susanna J. Dunachie, Derrick Crook, Andrew J. Pollard, Teresa Lambe, Philip Goulder, Neil G. Paterson, Mark A. Williams, David R. Hall, OPTIC Consortium, Christopher Conlon, Alexandra Deeks, John Frater, Lisa Frending, Siobhan Gardiner, Anni Jämsén, Katie Jeffery, Tom Malone, Eloise Phillips, Lucy Rothwell, Lizzie Stafford, ISARIC4C Consortium, Elizabeth E. Fry, Jiandong Huo, Juthathip Mongkolsapaya, Jingshan Ren, David I. Stuart, Gavin R. Screaton

**Affiliations:** 1 Wellcome Centre for Human Genetics, Nuffield Department of Medicine, University of Oxford, Oxford, UK; 2 Division of Structural Biology, Nuffield Department of Medicine, University of Oxford, The Wellcome Centre for Human Genetics, Oxford, UK; 3 Chinese Academy of Medical Science (CAMS) Oxford Institute (COI), University of Oxford, Oxford, UK; 4 Diamond Light Source Ltd, Harwell Science & Innovation Campus, Didcot, UK; 5 Oxford University Hospitals NHS Foundation Trust, Oxford, UK; 6 Peter Medawar Building for Pathogen Research, Oxford, UK; 7 Nuffield Department of Clinical Neurosciences, University of Oxford, Oxford, UK; 8 Translational Gastroenterology Unit, University of Oxford, Oxford, UK; 9 Oxford Vaccine Group, Department of Paediatrics, University of Oxford, Oxford, UK; 10 Nuffield Department of Medicine, University of Oxford, Oxford, UK; 11 Viral Pseudotype Unit, Medway School of Pharmacy, University of Kent and Greenwich Chatham Maritime, Kent, UK; 12 NIHR Oxford Biomedical Research Centre, Oxford, UK; 13 Centre For Tropical Medicine and Global Health, Nuffield Department of Medicine, University of Oxford, Oxford, UK; 14 Mahidol-Oxford Tropical Medicine Research Unit, Department of Medicine, University of Oxford, Oxford, UK; 15 Department of Paediatrics, University of Oxford, Oxford, UK

**Keywords:** VoC, SARS-CoV-2, antibody escape, Omicron, COVID-19, variant, BA.4, BA.5

## Abstract

The Omicron lineage of SARS-CoV-2, which was first described in November 2021, spread rapidly to become globally dominant and has split into a number of sublineages. BA.1 dominated the initial wave but has been replaced by BA.2 in many countries. Recent sequencing from South Africa’s Gauteng region uncovered two new sublineages, BA.4 and BA.5, which are taking over locally, driving a new wave. BA.4 and BA.5 contain identical spike sequences, and although closely related to BA.2, they contain further mutations in the receptor-binding domain of their spikes. Here, we study the neutralization of BA.4/5 using a range of vaccine and naturally immune serum and panels of monoclonal antibodies. BA.4/5 shows reduced neutralization by the serum from individuals vaccinated with triple doses of AstraZeneca or Pfizer vaccine compared with BA.1 and BA.2. Furthermore, using the serum from BA.1 vaccine breakthrough infections, there are, likewise, significant reductions in the neutralization of BA.4/5, raising the possibility of repeat Omicron infections.

## Highlights

• BA.4/5 resist neutralization by triple-dosed vaccinee serum more than BA.1 and BA.2
• BA.1 vaccine breakthrough serum shows reduced neutralization of BA.4/5
• Activity of SARS-CoV-2 therapeutic antibodies against BA.4/5 is reduced
• L452R and F486V mutations both make major contributions to BA.4/5 escape

## Graphical abstract

**Figure:**
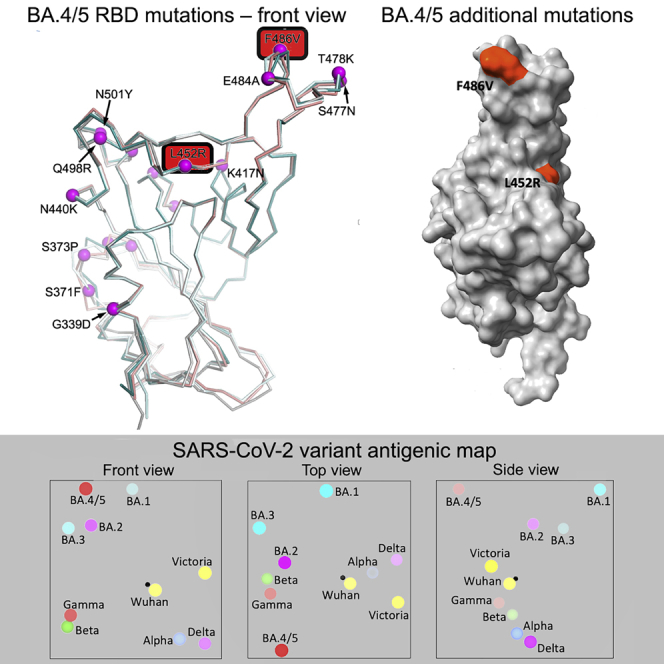

## Teaser

SARS-CoV-2 Omicron BA.4 and BA.5 sublineages bear mutations that lead to their reduced neutralization by sera from triple-vaccinated individuals when compared with the more recent BA.1 and BA.2. Importantly, sera from individuals with breakthrough BA.1 infections also show reduced neutralization, suggesting that repeat Omicron infections are likely in the population.

## Introduction

SARS-CoV-2 emerged in Wuhan in late 2019 to rapidly cause a pandemic. It is now estimated to have infected over half a billion people and caused over 6 million deaths (https://covid19.who.int/). Although SARS-CoV-2 RNA polymerase possesses some proofreading ability, there has been a rapid evolution of the viral sequence. Because of the scale of the pandemic, it is estimated that all single-point mutations in the large SARS-CoV-2 genome will be generated every day (Sender et al., 2021). Most mutations will be silent, deleterious, or of little consequence; however, a few may give the virus an advantage leading to rapid natural selection (Domingo, 2010). Many thousands of individual mutations have been described, and about a year after the outbreak started, strains began to emerge containing multiple mutations, particularly in the spike (S) gene. Several of these have been designated variants of concern (VoCs) (https://www.cdc.gov/coronavirus/2019-ncov/variants/variant-classifications.html) and have led to successive waves of infection: first, Alpha (Supasa et al., 2021), second, Delta (Liu et al., 2021a), and then Omicron (Dejnirattisai et al., 2022) spread globally, becoming the dominant variants. Alongside these, Beta (Zhou et al., 2021) and Gamma (Dejnirattisai et al., 2021b) caused large regional outbreaks in Southern Africa and South America, respectively, but did not dominate globally. As of April 29^th^, over 2.5 million cases of Omicron (BA.1 and BA.2) have been reported in the UK alone (https://www.gov.uk/government/publications/covid-19-variants-genomically-confirmed-case-numbers/variants-distribution-of-case-data-29-april-2022#omicron), and although the disease is less severe, particularly in vaccinated individuals, the scale of the outbreak has still led to a large number of deaths (Nealon and Cowling, 2022).

S is the major surface glycoprotein on SARS-CoV-2 and assembles into extended transmembrane anchored trimers (Walls et al., 2020; Wrapp et al., 2020), which give virions their characteristic spiky shape. S is divided into N-terminal S1 and C-terminal S2 regions. S1 contains the N-terminal domain (NTD) and receptor-binding domain (RBD). A small 25 amino acid (aa) patch at the tip of the RBD is responsible for interaction with the cellular receptor angiotensin-converting enzyme 2 (ACE2) (Lan et al., 2020). Following ACE2 binding, S1 is cleaved and detached, whereas S2 undergoes a major conformational change to expose the fusion loop, which mediates the fusion of viral and host membranes, allowing the viral RNA to enter the host cell cytoplasm and commence the replicative cycle (Walls et al., 2017).

S is the major target for neutralizing antibodies, and studies by a number of groups have isolated panels of monoclonal antibodies from infected or vaccinated volunteers (Barnes et al., 2020; Dejnirattisai et al., 2021a; Yuan et al., 2020a). Potently neutralizing antibodies are largely confined to three sets of sites on S1. The first is within the NTD (Cerutti et al., 2021; Chi et al., 2020); these antibodies do not block ACE2 interaction, and their mechanism of action is still not well determined. The second region of binding is on or in close proximity to the ACE2 binding surface of the RBD; most potently neutralizing antibodies bind this region and prevent the interaction of S with ACE2 on the host cell, blocking infection (Dejnirattisai et al., 2021a; Yuan et al., 2020a). Finally, some potent antibodies bind the RBD but do not block ACE2 binding, exemplified by mAb S309, which binds in the region of the N-linked glycan at position 343 (Pinto et al., 2020), these antibodies may function to destabilize the S-trimer (Huo et al., 2020b; Yuan et al., 2020b; Zhou et al., 2020).

Although mutations in the VoC are spread throughout S, there are particular hotspots in the NTD and RBD, exactly where potent neutralizing antibodies bind, and they are likely being driven by escape from the antibody response following natural infection or vaccination. Mutation of the ACE2 interacting surface may also give an advantage by increasing ACE2 affinity for S or by possibly altering receptor tropism (Zahradník et al., 2021). Increased ACE2 affinity has been found in VoC compared with ancestral strains (Dejnirattisai et al., 2021b; Liu et al., 2021a; Supasa et al., 2021; Zhou et al., 2021), potentially conferring a transmission advantage, but affinity is not increased in Omicron BA.1 (Dejnirattisai et al., 2022) and only marginally in BA.2 (Nutalai et al., 2022).

The initial Omicron wave was caused by the BA.1 strain, which, compared with ancestral strains, contains 30-aa substitutions, 6-aa deletions, and 3-aa insertions, which are largely clustered at the sites of interaction of potently neutralizing antibodies: the ACE2 interacting surface, around the N343 glycan, and in the NTD (Dejnirattisai et al., 2022). These changes cause large reductions in the neutralization titers of vaccine or naturally immune serum, leading to high levels of vaccine breakthrough infections and contributing to the intensity of the Omicron wave of infection (Dejnirattisai et al., 2022; McCallum et al., 2022).

**Figure 1:**
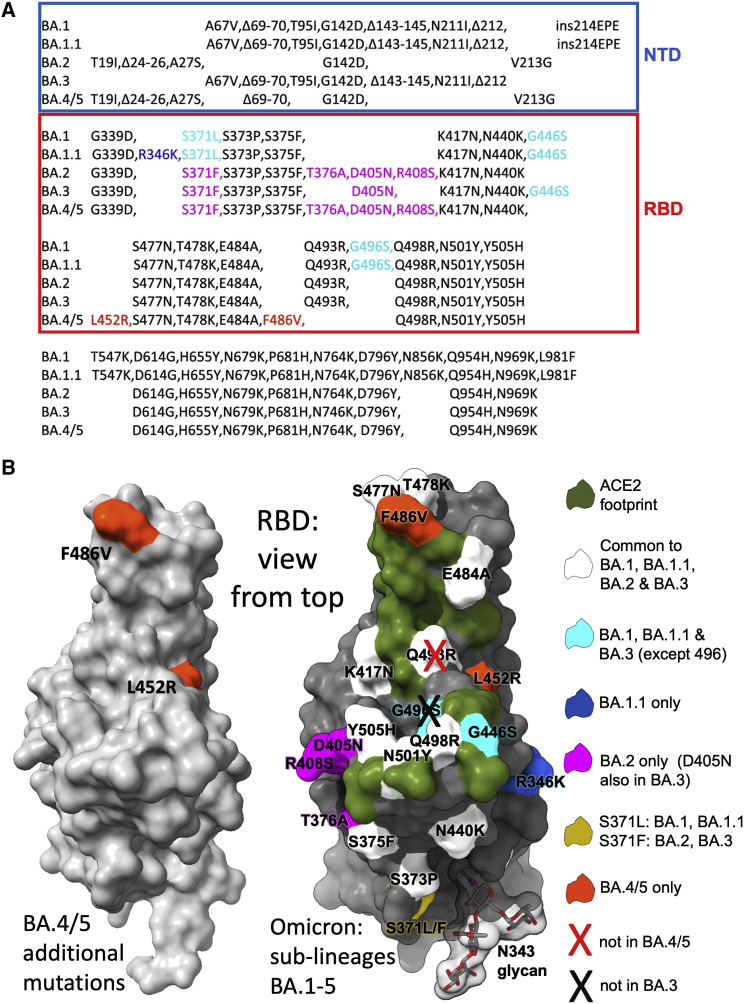
The Omicron sublineage compared with BA.4/5 (A) Comparison of S protein mutations of Omicron BA.1, BA.1.1, BA.2, BA.3, and BA.4/5 with NTD and RBD boundaries indicated. (B) Position of RBD mutations (gray surface with the ACE2 footprint in dark green). Mutations common to all Omicron lineages are shown in white (Q493R, which is reverted in BA.4/5, is shown with a cross), those common to BA.1 and BA.1.1 in cyan, those unique to BA.1.1 in blue, and those unique to BA.2 in magenta. Residue 371 (yellow) is mutated in all Omicron viruses but differs between BA.1 and BA.2. The N343 glycan is shown as sticks with a transparent surface.

A number of Omicron sublineages have been described. BA.2 and BA.3 were reported at about the same time as BA.1 and are highly related but contain some unique changes in S (Figure 1A), while another sublineage BA.1.1, which contains an additional R346K mutation, also emerged (Nutalai et al., 2022). The BA.2 strain, which possesses a small transmission advantage, has become globally dominant. BA.3, reported in relatively few sequences compared with BA.1 and BA.2, appears to be a mosaic of BA.1 and BA.2 changes (with 3 differences in the RBD compared with BA.1 and 3 differences compared with BA.2). Cases of BA.2 infection following BA.1 are not thought to be common due to good levels of cross-neutralizing antibodies following vaccination (Nutalai et al, 2022, https://www.who.int/news/item/22-02-2022-statement-on-omicron-sublineage-ba.2).

In early April 2022, two new Omicron lineages were reported from Gauteng in South Africa and designated BA.4 and BA.5 (https://assets.publishing.service.gov.uk/government/uploads/system/uploads/attachment_data/file/1067672/Technical-Briefing-40-8April2022.pdf). These have become dominant in Gauteng and look to be fueling a new wave of infection in South Africa, with some international spread. BA.4 and BA.5 (from here on referred to as BA.4/5) have identical S sequences and appear to have evolved from BA.2. They contain additional mutations in the RBD, in particular, the reversion mutation R493Q (Q493 is found in ancestral strains), together with mutations L452R and F486V (Figure 1A).

Here, we report the antigenic characterization of BA.4/5 compared with the other Omicron sublineages (for completeness, we also report data on BA.3, although this is of less concern). We find that the neutralization of BA.4/5 by triple-dosed vaccine serum is reduced compared with BA.1 and BA.2. We also see reductions in titers against BA.4/5 compared with BA.1 and BA.2 in the sera from individuals who had suffered vaccine breakthrough BA.1 infections. The neutralization of the Omicron lineage by a panel of recently derived potent Omicron-specific mAbs raised following vaccine breakthrough BA.1 infection (Nutalai et al., 2022) is reduced: 10/28 are completely knocked out against BA.4/5, while several others suffer large reductions in activity compared with the other Omicron lineages. We corroborate the neutralization results with a biophysical analysis of binding and provide structure-function explanations for mAb failure against BA.4/5 with the changes at residues 452 and 486, both of which cause serious impact. Finally, we measure the affinity of the BA.4/5 RBD for ACE2 and find that it is higher than earlier Omicron strains BA.1 and BA.2.

## Results

### The Omicron lineages BA.4/5

BA.4 and BA.5 S sequences are identical and closely related to BA.2 (sequence diversity in Omicron S is shown in Figure 1A). Compared with BA.2, BA.4/5 has residues 69 and 70 deleted and contains 2 additional substitutions in the RBD: L452R and F486V. Finally, BA.4/5 lacks the Q493R change seen in BA.1 and BA.2, reverting to Q493 as in the Victoria/Wuhan strain.

The 2 additional mutations in the RBD are of most concern in terms of antibody escape: L452R is a chemically radical change and is one of the pair of changes in Delta RBD (the other, T478K, is already found in the Omicron lineage), and L452R is also found in Epsilon and the recently reported Omicron BA.2.11 (https://www.who.int/activities/tracking-SARS-CoV-2-variants). Mutation F486L was found in the sequences of SARS-CoV-2 isolated from Mink early in the pandemic. F486 is also a site of escape mutations to several mAbs (Gobeil et al., 2021), and F486I was noted during SARS-CoV-2 evolution in an immunocompromised individual (Clark et al., 2021). The change F486V in BA.4/5 also causes a reduction in the bulk of the hydrophobic side chain as in F486L but is more significant. Both residues 452 and 486 lie close to the edge of the ACE2 interaction surface (Figure 1B) and, together with the reversion to ancestral sequence Q493, which lies within the ACE2 footprint, have the potential to modulate ACE2 affinity and the neutralizing capacity of the vaccine or naturally acquired serum. The L452R and F486V mutations are likely to cause more antibody escape, whereas the reversion at 493 may reduce the escape from the responses to earlier viruses.

**Figure S1:**
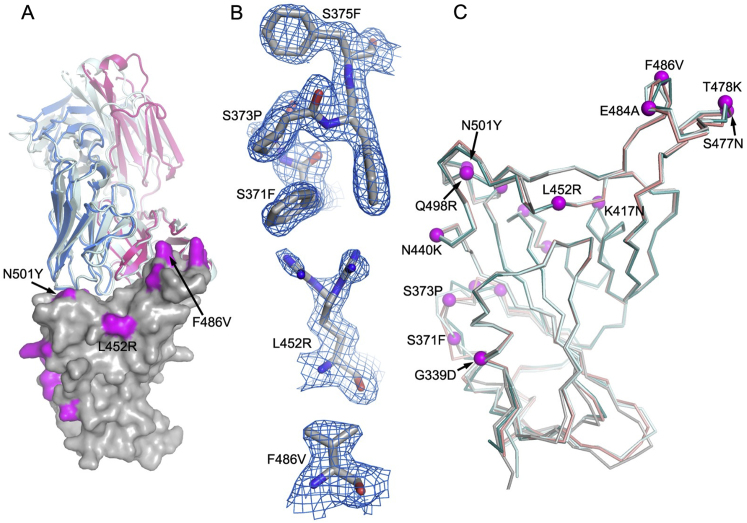
Overall structure of BA.4 RBD/Beta-27 complex, related to Table S1 and STAR Methods (A) Comparison of BA.4 RBD/Beta-27 (the bound nanobody C1 is omitted for clarity) with Beta RBD/Beta-27 (PDB: 7PS1) by overlapping the RBDs. The RBD is shown as a gray surface with mutation sites highlighted in magenta. The heavy chain and light chain are drawn as red and blue ribbons, respectively, for the BA.4 RBD/Beta-27 complex; Beta-27 in the Beta RBD complex is colored in pale cyan. The overall binding modes of the Fab in the two complexes are very similar, although there are some differences in the side-chain orientations at the interface, such as R403, N417, and Q493 of the RBD. The light-chain CDR3 becomes flexible in the BA.4 complex. (B) Electron density maps. Residues 371–375 that carry the S371L/F, S373P, and S375F mutations are flexible in the BA.1 and BA.2 RBD/Fab complexes (PDB: 7ZF3 and 7ZF8) but are well ordered in this high BA.4/5 resolution structure (top panel). L452R has double conformation (middle panel), and F486V has a well-defined density (bottom panel). (C) Comparison of the RBD of BA.4 (gray) with those of BA.1 (teal), BA.2 (cyan), and Beta (salmon). Mutation sites in BA.4 are shown as magenta spheres.

To verify structural inferences, the crystal structure of BA.4/5 RBD was determined at 1.9 Å as a ternary complex with a neutralizing Fab and nanobody (Table S1; Figure S1). This confirmed that the structure of the BA.4/5 RBD is very similar to that of other variants, although the residue 371–375 region, which is a hotspot of Omicron-specific mutations, is unusually well ordered and the tip of the arginine side chain of L452R is found in two conformations (Figure S1).

### Neutralization of BA.4/5 by vaccine serum

**Figure 2:**
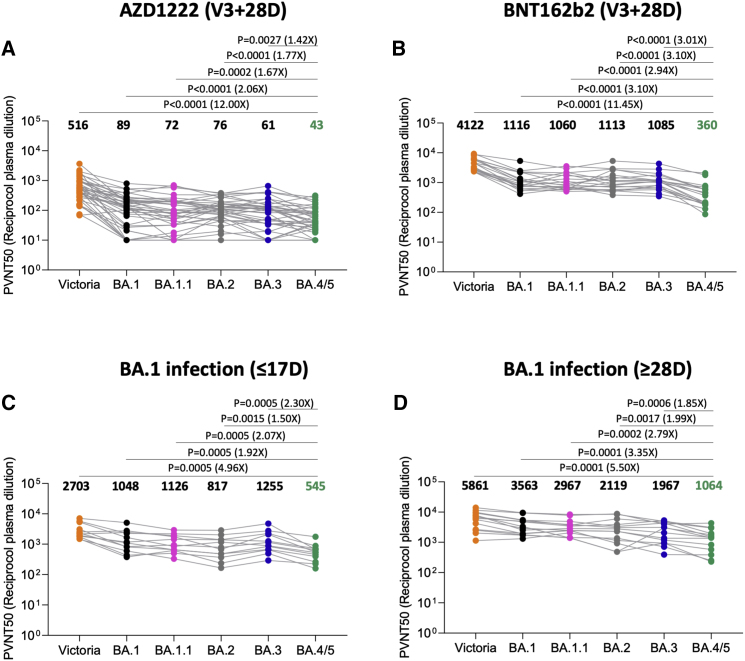
Pseudoviral neutralization assays of BA.4/5 by vaccine and BA.1 immune serum (A and B) IC50 values for the indicated viruses using serum obtained from vaccinees 28 days following their third dose of vaccine (A) AstraZeneca AZD1222 (n = 41) or (B) 4 weeks after the third dose of Pfizer BNT162b2 (n = 19). (C and D) Serum from volunteers suffering breakthrough BA.1 infection taken (C) early, i.e., ≤17 days from symptom onset (median 12 days) n = 12 and (D) late, i.e., ≥28 days from symptom onset (median 45 days) n = 14. Comparison is made with neutralization titers to Victoria an early pandemic strain, BA.1, BA.1.1, BA.2, and BA.3. Geometric mean titers are shown above each column. The Wilcoxon matched-pairs signed-rank test was used for the analysis, and two-tailed p values were calculated.

We constructed a panel of pseudotyped lentiviruses (Di Genova et al., 2020) expressing the S gene from the Omicron sublineages BA.1, BA.1.1, BA.2, BA.3, and BA.4/5 together with the early pandemic Wuhan-related strain, Victoria, used as a control. Neutralization assays were performed using serum obtained 28 days following a third dose of the Oxford-AstraZeneca vaccine AZD1222 (n = 41) (Flaxman et al., 2021) or Pfizer-BioNtech vaccine BNT162b2 (n = 19) (Cele et al., 2022a; Figures 2A and 2B). For AZD1222, neutralization titers for BA.4/5 were reduced 2.1-fold compared with BA.1 (p < 0.0001) and 1.8-fold compared with BA.2 (p < 0.0001). For BNT162b2, neutralization titers were reduced 3.1-fold (p < 0.0001) and 3.1-fold (p < 0.0001) compared with BA.1 and BA.2, respectively. These reductions in titers may reduce the effectiveness of the vaccines at preventing infection, particularly at longer time points, as antibody titers naturally wane, although it would be expected that protection would remain against severe disease.

### Neutralization of BA.4/5 by serum from breakthrough BA.1 infection

Early in the Omicron outbreak when BA.1 predominated, we recruited vaccinated volunteers who had suffered PCR-confirmed SARS-CoV2 infection—most were sequence-confirmed BA.1 infections or contacts of BA.1 confirmed cases, and all of the infections were mild. Early samples (n = 12, 9F and 3M; median age is 26; and median time since vaccine is 141 days) were taken ≤17 days from symptom onset (median is 12 days), and later samples (n = 14, 7F and 7M; median age is 23; and median time since vaccine is 111 days) were taken ≥28 days following symptom onset (median is 45 days). All cases had been vaccinated, all but 2 had received 2 doses, and 3 of the late convalescent cases received a third dose of vaccine following Omicron infection. Pseudoviral neutralization assays were performed against the panel of pseudoviruses described above (Figures 2C and 2D).

As we have previously described, BA.1 infection following vaccination leads to a broad neutralizing response, with high titers to all the VoC, which is boosted at later time points (Nutalai et al., 2022). Neutralization titers against BA.4/5 were significantly less than those against BA.1 and BA.2. At the early time point, BA.4/5 titers were reduced 1.9- (p = 0.0005) and 1.5-fold (p = 0.0015) compared with BA.1 and BA.2, respectively. At the later point, BA.4/5 titers were reduced 3.4- (p = 0.0001) and 2-fold (p = 0.0017) compared with BA.1 and BA.2, respectively.

Thus, BA.4/5 shows a degree of immune escape from the vaccine/BA.1 response when compared with BA.1 and BA.2. These samples were all taken reasonably close to the time of infection meaning that further waning in the intervening months may render individuals susceptible to reinfection with BA.4/5.

### Escape from monoclonal antibodies by BA.4/5

**Figure 3:**
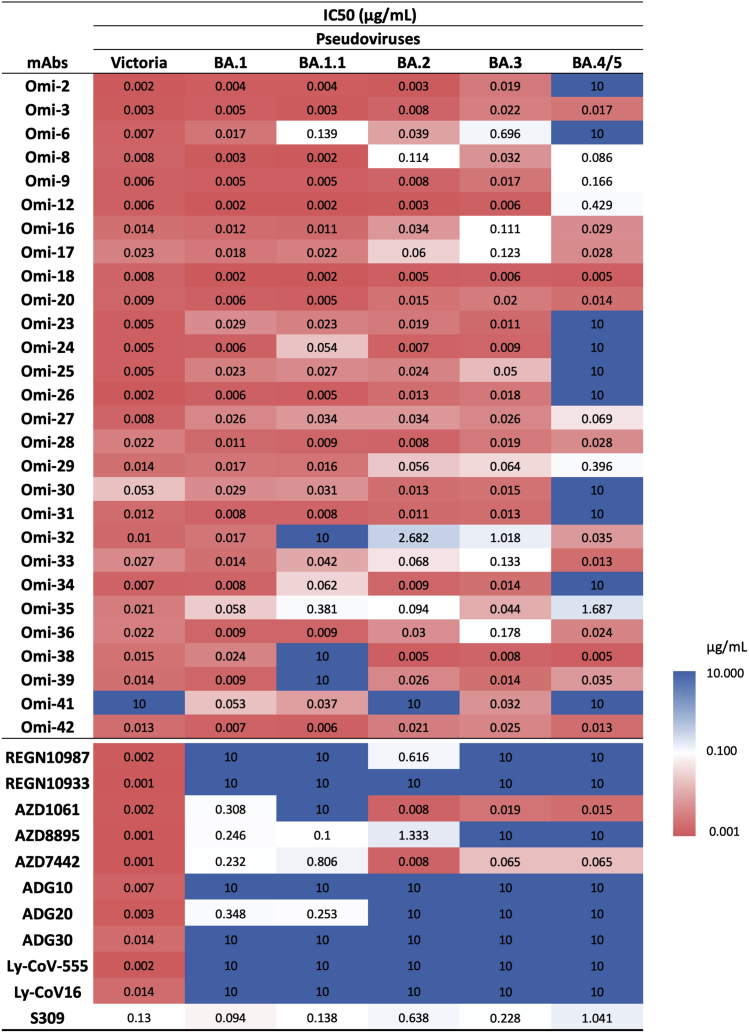
IC50 values for Omicron and commercial mAbs See also Figures S2, S3, S4, and S5.

**Figure S2:**
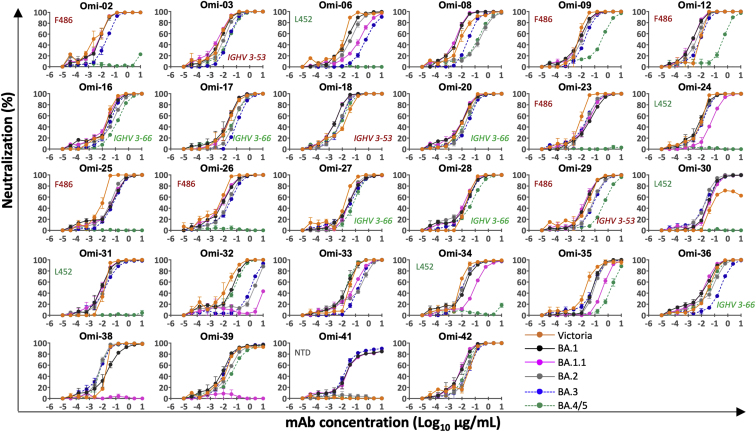
Pseudoviral neutralization assays against Omicron monoclonal antibodies, related to Figure 3 where IC50 titers are shown Neutralization curves for a panel of 28 monoclonal antibodies made from samples taken from vaccinees infected with BA.1. Titration curves for BA.4/5 are compared with Victoria, BA.1, BA.1.1, BA.2, and BA.3, and mAbs we propose to be affected by the L452R and F486V mutations are indicated as are those belonging to the IGVH3-53/66 gene families.

We have recently reported a panel of potent human mAb generated from cases of Omicron breakthrough infection (Nutalai et al., 2022). For the 28 most potent mAbs (BA.1 IC50 titers <100 ng/mL), we used pseudoviral assays to compare BA.4/5 neutralization with the neutralization of BA.1, BA.1.1, BA.2, and BA.3 (Figures 3 and S2). The neutralization of BA.4/5 was completely knocked out for 10/28 mAbs. Four further mAbs (Omi-09, 12, 29, and 35) showed >5-fold reduction in the neutralization titer of BA.4/5 compared with BA.2. All of these antibodies interact with the RBD, with the exception of Omi-41, which binds the NTD and specifically neutralizes BA.1, BA.1.1, and BA.3 but not BA.2 or BA.4/5 (for unknown reasons, Omi-41 can neutralize wild-type (WT) Victoria virus but not Victoria pseudovirus) (Nutalai et al., 2022).

## Sensitivity to L452R

We have previously reported that Omi-24, 30, 31, 34, and 41 show complete knockout of neutralizing activity against Delta, with Omi-06 showing a severe knockdown of activity (Nutalai et al., 2022). Since BA.1 and BA.2 harbor only one (T478K) of the 2 Delta RBD mutations, while BA.4/5 also harbor L452R, we would expect all five of these L452-directed mAbs to be knocked out on BA.4/5. This is indeed observed (Figures 3 and S2). Omi-41 also fails to neutralize, which is attributed to the differences in mutations in the NTD (Figure 1A).

**Figure S3:**
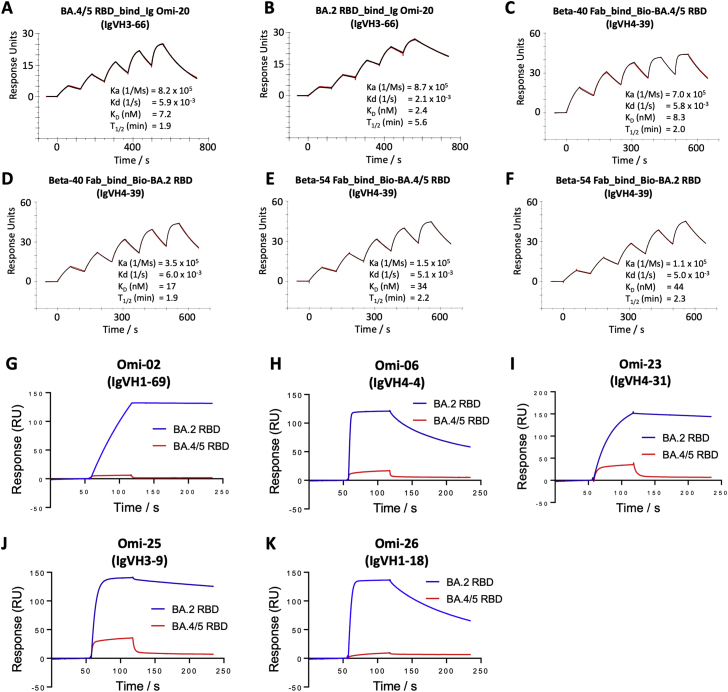
Surface plasmon resonance (SPR) analysis of the interaction between BA.2 or BA.4/5 RBD and selected mAbs, related to Figure 3 (A–F) Sensorgrams (red: original binding curve; black: fitted curve) showing the interactions between BA.2 or BA.4/5 RBD and selected mAbs, with kinetics data shown. (G–K) Binding of BA.4/5 RBD is severely reduced compared with that of BA.2, so the binding could not be accurately determined, as shown by a single injection of 200 nM RBD over sample flow cells containing the mAb indicated.

**Figure 4:**
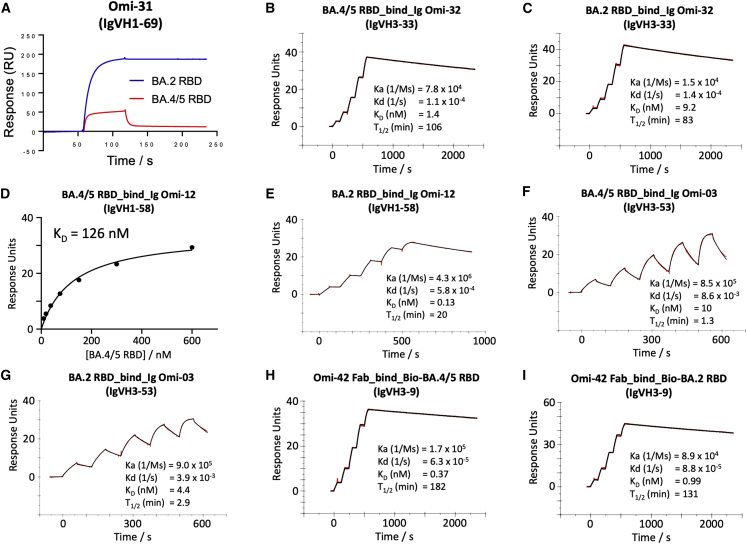
Surface plasmon resonance (SPR) analysis of the interaction between BA.2 or BA.4/5 RBD and selected mAbs (A) Binding of BA.4/5 RBD is severely reduced compared with that of BA.2, so the binding could not be accurately determined, as shown by a single injection of 200 nM RBD over sample flow cells containing IgG Omi-31. (B, C, and E–I) Sensorgrams (red: original binding curve; black: fitted curve) showing the interactions between BA.2 or BA.4/5 RBD and selected mAbs, with kinetics data shown. (D) Determination of the affinity of BA.4/5 RBD to Omi-12 using a 1:1 binding equilibrium analysis. See also Figures 3 and S3.

**Figure 5:**
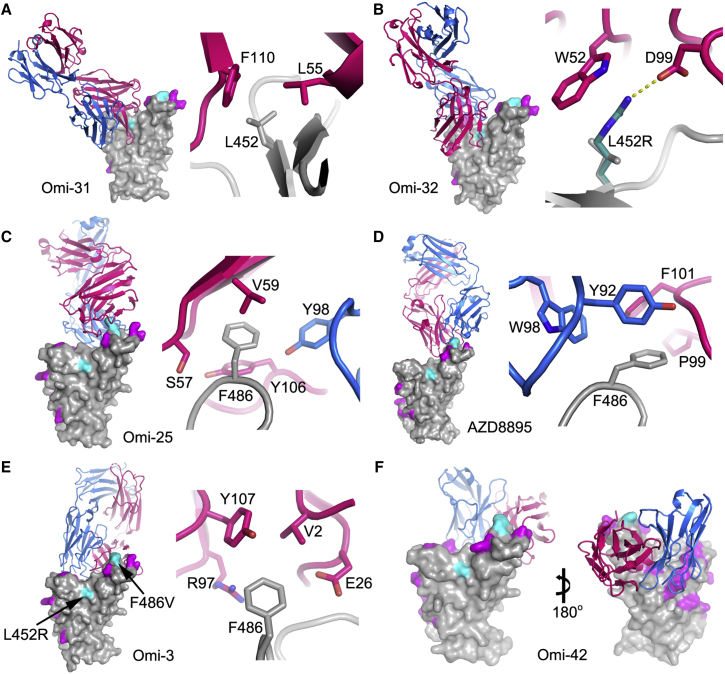
Interactions between mAb and BA.4/5 mutation sites Overall structure (left panel) and interactions (≤4 Å) with BA.4/5 mutation sites (right panel) for (A) BA.1-RBD/Omi-31 (PDB: 7ZFB), (B) BA.1-RBD/Omi-32 (PDB: 7ZFE), (C) BA.1-RBD/Omi-25 (PDB: 7ZFD), (D) Wuhan-RBD/AZD8895 (PDB: 7L7D), (E) BA.1-RBD/Omi-3 (PDB: 7ZF3), and (F) BA.1-RBD/Omi-42 (PDB: 7ZR7) complexes. In the left panels, RBD is shown as surface representation, with BA.4/5 mutation sites highlighted in magenta and the additional two mutation sites of BA.4/5 at 452 and 486 in cyan and Fab LC as blue and HC as red ribbons. In the right panel, side chains of RBD, Fab HC, and LC are drawn as gray, red, and blue sticks, respectively. In (B), the L452R mutation (cyan sticks) is modeled to show that a salt bridge to D99 of CDR-H3 may be formed (yellow broken sticks). (F) shows that the Fab of Omi-42 does not contact either of the two BA.4/5 mutation sites. See also Figure S1.

To confirm that the neutralization effects observed are directly attributable to alterations in RBD interactions, we also performed binding analyses of selected antibodies to BA.4/5 and BA.2 RBDs by surface plasmon resonance (SPR) (Figures 4 and S3). Omi-31 was chosen as the representative of the set of L452R sensitive antibodies, and as expected, the binding is severely affected (Figure 4A). Since we have detailed information on the interaction of several Omicron responsive antibodies with the RBD, including Omi-31, we modeled the BA.4/5 RBD mutations in the context of known structures for Omicron Fabs complexed with BA.1 or Delta RBDs (Dejnirattisai et al., 2022; Nutalai et al., 2022; Figure 5). The Omi-31 complex is shown in Figure 5A, and it reveals that L452 is tucked neatly into a hydrophobic pocket, which is unable to accommodate the larger positively charged arginine in BA.4/5 and Delta without major conformational changes.

### L452R enhancement of binding

Omi-32 shows 77-fold enhanced neutralization of BA.4/5 compared with BA.2. Kinetic analysis of Fab binding to the RBDs suggests that this is mainly achieved by a 5-fold increase in the on rate of binding (Figures 4B and 4C). This could be explained by the arginine at 452 making a salt bridge to residue 99 of the heavy-chain (HC) CDR3 (Figure 5B). It is possible that electrostatic changes enhance on rate by electrostatic steering of the incoming antibody.

## Sensitivity to F486V

Extending the logic used to understand Delta sensitivity, the remaining antibodies affected by BA.4/5 > BA.2, but which retain activity against Delta, namely Omi-02, 09, 12, 23, 25, 26, and 29, are likely sensitive to the F486V change. The binding sensitivity was confirmed by SPR analysis of Omi-12, a VH1-58 family member, which, like AZD 8895 (below), binds over F486 (Nutalai et al., 2022; Figures 4D and 4E) and showed an almost 1,000-fold reduction in affinity to BA.4/5.

Another example of the structural basis of sensitivity to F486V is provided by Omi-25, which shows reduced binding and no neutralizing activity against BA.4/5 (Figures 3 and S3J). The Omi-25 complex shows that the phenylalanine side chain acts as a binding hotspot, nestled in a hydrophobic cavity making favorable ring-stacking interactions with Y106 of the HC CDR3 (Figure 5C).

### The activity of commercial antibodies against BA.4/5

**Figure S4:**
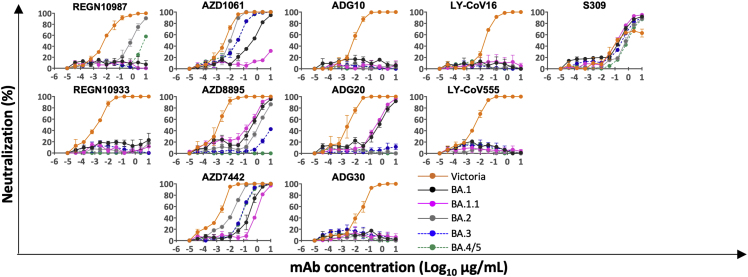
Pseudoviral neutralization assays against commercial monoclonal antibodies, related to Figure 3 where IC50 titers are shown Pseudoviral neutralization assays with mAbs developed for human use.

We tested a panel of antibodies that have been developed for therapeutic/prophylactic use against BA.4/5 (Figures 3 and S4). Many of these antibodies have already suffered severe reductions or knockout of activity against BA.1, BA.1.1, or BA.2. For AstraZeneca AZD1061, activity against BA.4/5 was similar to that against BA.2 (<2-fold reduction), while for AZD8895, residual activity against BA.2 was knocked out. The activity of the combination of both antibodies in AZD7442 (Dong et al., 2021) was reduced 8.1-fold compared with BA.2. The residual activity of REGN10987 (Weinreich et al., 2021) against BA.2 was further reduced on BA.4/5; likewise, residual BA.1 neutralizing activity was knocked out for ADG20 (Yuan et al., 2022) on BA.4/5. For S309 (VIR-7831/7832) (Sun and Ho, 2020), the activity against BA.4/5 was 1.6-fold reduced compared with BA.2.

**Figure S5:**
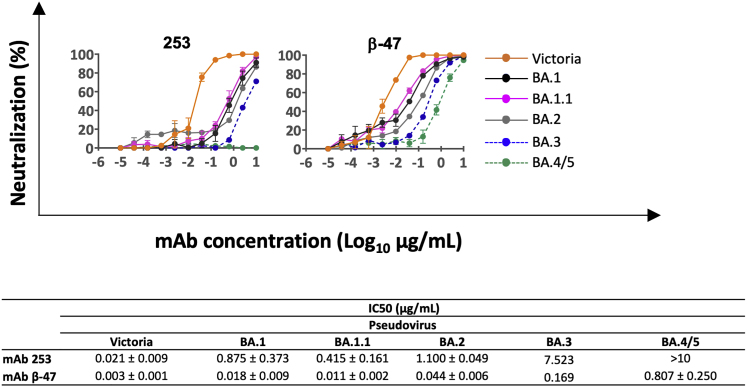
Neutralization curves for IGVH1-58 mAb, related to Figure 3 Pseudoviral neutralization curves for early pandemic mAb 253 (Dejnirattisai et al., 2021a) and Beta-47 (Liu et al., 2021b) against Victoria and the panel of Omicron lineage constructs.

These effects can be rationalized by reference to the way the antibodies interact with the RBD; for instance, in the case of AZD8895 (an IGVH1-58 genotype mAb, Figure 5D), F486 forms a hydrophobic interaction hotspot, which will be abrogated by the mutation to a much smaller valine side chain. Antibody residues involved in the interactions with F486 are highly conserved among this genotype of mAbs, including Omi-12, 253, and Beta-47 (Nutalai et al., 2022; Dejnirattisai et al., 2021a; Liu et al., 2021b), explaining the severe effect of the F486V mutation on neutralization of these mAbs (Figures 3 and S5).

### Systematic themes in mAb interactions

Both Omi-3 (a representative of the IGVH3-53 gene family) and AZD8895 (IGVH1-58) make contact with F486. While the F486V mutation has little effect on Omi-3 (Figures 3, 4F, 4G, and 5E), it seriously reduces the neutralization of AZD8895 and other IGVH1-58 mAbs, e.g., Omi-12 (Figures 3, 4D, 4E, and 5D). It is notable that whereas the numerous Omi series antibodies belonging to the closely related IGVH3-53 and IGVH3-66 gene families (9/28 in total Figure S2) are almost entirely resilient to the BA.4/5 changes, the large majority of antibodies from these gene families elicited against earlier variants are knocked out on BA.1 and BA.2 (Nutalai et al., 2022), consistent with selection of a subset of antibodies by breakthrough Omicron infection that is insensitive to the further BA.4/5 mutations.

The effects on antibodies with broadly similar epitopes can vary dramatically, and this is equally true for antibodies, which have 452 or 486 central to their binding footprint. Thus, Omi-31 (IGVH1-69) and Omi-32 (IGVH3-33) both bind in front of the right shoulder with their CDR-H3 positioned close to 452; while the activity of Omi-31 is abolished by L452R (as detailed above), Omi-32 is markedly enhanced (Figures 3, 5A, 5B, and S2). Similarly, Omi-25 and Omi-42 both belong to the IGVH3-9 gene family, and their footprints are in the 486 region (Figures 5C and 5F). Omi-25 contacts F486, and thus, the neutralization of BA.4/5 is abolished. By contrast, Omi-42 does not contact either of the mutation sites, and neutralization is fully retained for BA.4/5 (Figures 3, 4H, 4I, and 5F).

### ACE2 RBD affinity

**Figure 6:**
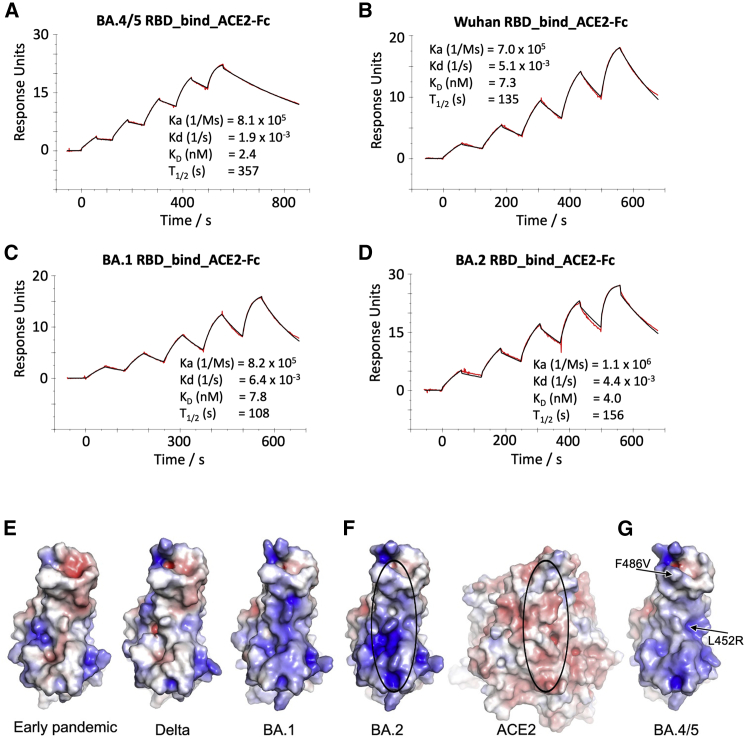
ACE2 RBD affinity (A–D) SPR sensorgrams showing ACE2 binding of BA.4/5 RBD (A) in comparison with binding to ancestral (Wuhan) (B), BA.1 (C), and BA.2 RBD (D). The data for Wuhan, BA.1, and BA.2 have been reported previously in Nutalai et al. (2022). (E–G) Electrostatic surfaces, (E) from left to right, early pandemic, Delta, and BA.1 RBD. (F) Open book view of BA.2 RBD and ACE2 of the BA.2 RBD/ACE2 complex (PDB: 7ZF7) and (G) BA.4/5 RBD (PDB: 7ZXU). The lozenges on ACE2 and RBD show the interaction areas.

We measured the affinity of BA.4/5 RBD for ACE2 by SPR (Figures 6A–6D). The affinity of BA.4/5 RBD was increased compared with the ancestral virus (Wuhan), BA.1, and BA.2 (approximately 3-, 3-, and 2-fold, respectively [BA.4/5/ACE2 KD = 2.4 nM]) (Dejnirattisai et al., 2022; Nutalai et al., 2022), which is mainly attributed to an increase in binding half-life. Modeling of the ACE2/RBD complex suggests that the bulk of this effect comes from the electrostatic complementarity between ACE2 and the RBD contributed by the L452R mutation (Figures 6E–6G).

### Antigenic cartography

**Figure 7:**
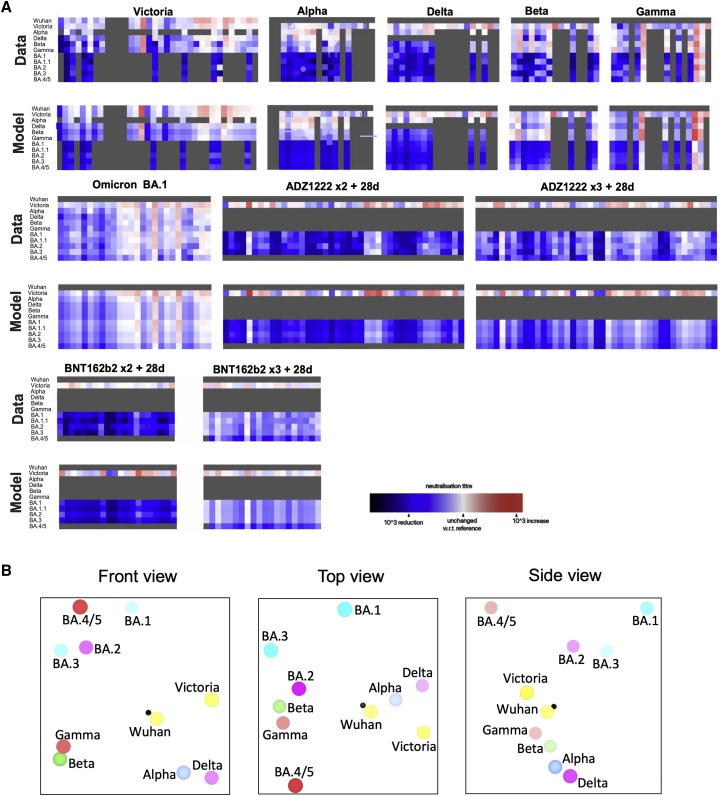
Antigenic mapping (A) Neutralization data and model (log titer values) used to calculate antigenic maps in (B). Columns represent sera collected from inoculated volunteers or infected patients. Rows are challenge strains: Victoria, Alpha, Delta, Beta, Gamma, BA.1, BA1.1, BA.2, BA.3, and BA.4/5 in order. Values are colored according to their deviation from the reference value. The reference value is calculated on a serum-type basis as the average of neutralization titers from the row that gives this the highest value. (B) Orthogonal views of the antigenic map showing BA.4/5 in the context of the positions of previous VoC and BA.1, BA.1.1, BA.1, and BA.2, calculated from pseudovirus neutralization data. Distance between two positions is proportional to the reduction in neutralization titer when one of the corresponding strains is challenged with a serum derived by infection by the other. No scale is provided since the figures are projections of a three-dimensional distribution; however, the variation can be calibrated by comparison with (i) BA.1 to BA.2, which is 2.93× reduced, and (ii) BA.2 to BA.4/5, which is 3.03× reduced. The third dimension may be inferred by fading of the colors with greater distance from the viewer.

The neutralization data above has been used to place BA.3 and BA.4/5 on an antigenic map. We repeated the method used for the analysis of the Delta and Omicron variants (Liu et al., 2021a), where individual viruses were independently modeled allowing for serum-specific scaling of the responses (STAR Methods). The measured and modeled responses are shown in Figure 7A (with 1,551 observations and 340 parameters, the residual error is 23%). The results are best visualized in three dimensions (see Video S1), but 2D projections are shown in Figure 7B. This shows, as expected, that the Omicron sublineages are clustered together but well separated from early pandemic virus and earlier VoC. Among the Omicron cluster, BA.4/5 is the most distant from the pre-Omicron viruses; the distance between BA.4/5 and BA.2 is similar to that between BA.2 and BA.1.

Video S1. Antigenic landscape for SARS-CoV-2, related to Figure 6B

## Discussion

Following the emergence of SARS-CoV-2 in November 2019, a succession of SARS-CoV-2 viral variants have appeared with increased fitness; these variants have rapidly outcompeted the preceding strain and spread globally—the most recent, Omicron, appearing in late 2021.

Despite the availability of vaccines, the pandemic has not been brought under control, and through Omicron, infections are as high as ever. Although vaccines are effective at preventing severe disease, they are less effective at preventing transmission, particularly of the Omicron sublineages. The very high level of viral replication globally drives the accrual of mutations in the viral genome, and we are now seeing the assembly of dozens of individual changes in single viruses. Virus recombination, which was predicted, is now being detected, allowing the shuffling of complex genomes, such as XD (Delta/BA.1) and XE (BA.1/BA.2), which in the latter case, may be more transmissible (https://assets.publishing.service.gov.uk/government/uploads/system/uploads/attachment_data/file/1063424/Tech-Briefing-39-25March2022_FINAL.pdf).

How such large sequence jumps, such as that to the Omicron lineage, occur is not known. It has been suggested that these may be occurring in immunocompromised or HIV-infected cases, where chronic infections have been documented to last for many months or in some cases over a year. The selection of antibody escape mutations has been documented in such individuals (Cele et al., 2022b; Karim et al., 2021; Kemp et al., 2021), and successive rounds of replication, recombination, and perhaps reinfection may be responsible for the selection of the constellation of S mutations found in the Omicron lineage.

BA.4/5, the most recently reported Omicron sublineages, seem to be taking hold in South Africa and may spread globally to replace BA.2. Although highly related to BA.2, BA.4/5 contain the 69-70 deletion in the NTD that was also found in Alpha, BA.1, and BA.3, together with additional mutations in the RBD (L452R and F486V). Thus, BA.4/5 has assembled mutations at all of the previously described positions in the VoC Alpha (N501Y), Beta (K417N, E484K, N501Y), Gamma (K417T, E484K, N501Y), and Delta (L452R, T478K), the only difference being E484A in BA.4/5 rather than E484K found in Beta and Gamma.

Here, we report a greater escape from the neutralization of BA.4/5 compared with BA.1 and BA.2. Serum from triple vaccinated donors has ∼2- to 3-fold reduction in neutralization titers compared with the neutralization of BA.1 and BA.2. Additionally, serum from breakthrough BA.1 infections in vaccinees shows ∼2- to 3-fold reduction in neutralization titers to BA.4/5 compared with BA.1 and BA.2. These reductions are in good agreement with the reductions of BA.4 and BA.5 neutralization titers reported following BA.1 vaccine breakthrough infections (Khan et al., 2022). These data suggest that a further wave of Omicron infection, driven by BA.4/5, is likely, partly due to breakthrough of vaccine and naturally acquired immunity, although there is no evidence yet of increased disease severity.

Using a panel of potent mAbs generated from vaccinated individuals infected with BA.1, we show the importance of the two new RBD mutations in BA.4/5. The activity of many mAbs is either knocked out or severely impaired against BA.4/5 compared with BA.2. From the neutralization data on BA.4/5, compared with that on Delta, we have been able to impute the contribution of L452R and F486V, and by combining with SPR data, as well as previous mapping by bio-layer interferometry (BLI) competition matrices and detailed structural data (Nutalai et al., 2022), we are able to understand the basis of these effects on neutralization and show that the L452R and F486V mutations both make major contributions to BA.4/5 escape.

It is clear that the Omicron lineage, particularly BA.4/5, has escaped or reduced the activity of mAbs developed for clinical use, with most mAbs showing complete knockout of activity. AZD7442 still shows activity against BA.4/5 (65 ng/mL), but 65-fold less than the activity against Victoria, and S309 activity against BA.4/5 is 8-fold reduced compared with Wuhan with IC50 titers >1,000 ng/mL. The reduction in the neutralizing activity of S309 reported here using pseudoviruses is less than that for WT viruses and may be due to differences in the assay format; for instance, the IC50 for BA.2 using pseudovirus is 638 ng/mL, while we reported 5,035 ng/mL using a WT virus (Nutalai et al., 2022).

New monoclonals and combinations may be needed to plug the gap in activity and protect the extremely vulnerable and those unable to mount adequate vaccine responses. There is also a question about vaccines. All current vaccines use spike derived from the original virus isolated from Wuhan. Vaccines have been remarkably effective at reducing severe disease, and a triple dosing schedule has provided, at least in the short term, protection against Omicron. However, the prevention of transmission may become less effective as viruses evolve antigenically further from ancestral strains. Some argue for next-generation vaccines tailored to antigenically distant strains, such as Omicron, to give better protection, probably used in combination with boosters containing ancestral strains. While vaccination is unlikely to eliminate transmission, the combination of vaccines with boosting by natural infection will probably continue to protect the majority from severe disease.

Finally, it is impossible to say where SARS-CoV-2 evolution will go next, but it is certain that the virus will continue to drift antigenically. This may be a continuation along the Omicron lineage, or we may see a large jump to a completely new lineage, like the one from Delta to Omicron. The observation that of the 30-aa substitutions in BA.1, all but one was achieved by a single base change in the codon suggests that there remains plenty of antigenic space for SARS-CoV-2 to explore and the capacity for recombination, which has so far not been observed to have breakpoints within the major antigenic sites, could generate a more radical antigenic shift.

### Limitations of the study

One of the limitations of this study is that serum was obtained at early time points following vaccination or breakthrough infection, so titers are likely to wane thereafter. In addition, the true *in vivo* protection induced by vaccination may be underestimated using *in vitro* neutralization assays where complement, antibody-dependent cell-mediated cytotoxicity and T cell responses are not operative. It would also be interesting to look at BA.4/5 neutralization using serum from unvaccinated individuals who had suffered primary BA.1 infection where the degree of escape of BA.4/5 may be greater than that seen with the vaccine breakthrough BA.1 serum reported here.

## STAR★Methods

### Key resources table

**Table:** 

REAGENT or RESOURCE	SOURCE	IDENTIFIER
**Antibodies**		

Nanobody C1	Huo et al., 2020a	N/A
Fab	Dejnirattisai et al., 2021a	N/A
IgG	Dejnirattisai et al., 2021a; Liu et al., 2021b	N/A
EY6A mAb	Zhou et al., 2020	N/A
Regeneron mAbs	AstraZeneca	Cat#REGN10933, and REGN10987
AstraZeneca mAbs	AstraZeneca	Cat#AZD1061, AZD8895, and AZD7442
Vir mAbs	Adagio	Cat#S309
Lilly mAbs	Adagio	Cat#Ly-CoV555, and Cat#Ly-CoV16
Adagio mAbs	Adagio	Cat#ADG10, Cat#ADG20, and Cat#ADG30
28 mAbs generated from cases of Omicron breakthrough infection	Nutalai et al., 2022	N/A
Anti-c-Myc 9E10 antibody	Biolegend	Catt#626872

**Bacterial, virus strains, and yeast**		

DH5α bacteria	InVitrogen	Cat#18263012
Saccharomyces cerevisiae EBY100	ATCC	Cat#MYA-4941
E. coli clone 10G cells	Lucigen, USA	Cat#60117-1

**Biological samples**		

Serum from Pfizer-vaccinated individuals	University of Oxford	N/A
Serum from AstraZeneca-Oxford-vaccinated individuals	University of Oxford	N/A
Plasma from SARS-CoV-2 patients	John Radcliffe Hospital in Oxford UK, South Africa, and FIOCRUZ (WHO) Brazil	N/A

**Chemicals, peptides, and recombinant proteins**		

His-tagged SARS-CoV-2 RBD	Dejnirattisai et al., 2021a	N/A
His-tagged SARS-CoV-2/Omicron RBD	This paper	N/A
His-tagged SARS-CoV-2/Omicron BA.4 RBD	This paper	N/A
His-tagged SARS-CoV-2/Omicron BA.5 RBD	This paper	N/A
His-tagged SARS-CoV-2 RBD-62	Zahradník et al., 2021	N/A
His-tagged SARS-CoV-2 RBD N501Y	Supasa et al., 2021	N/A
His-tagged SARS-CoV-2 RBD K417N, E484K, N501Y	Zhou et al., 2021	N/A
His-tagged SARS-CoV-2 RBD K417T, E484K, N501Y	Dejnirattisai et al., 2021b	N/A
His-tagged SARS-CoV-2 RBD L452R, T478K	Liu et al., 2021a	N/A
His-tagged human ACE2	Liu et al., 2021a	N/A
Human ACE2-hIgG1Fc	Liu et al., 2021a	N/A
His-tagged 3C protease	Libby et al., 1988	N/A
Phosphate buffered saline tablets	Sigma-Aldrich	Cat#P4417
Dulbecco’s Modified Eagle Medium, high glucose	Sigma-Aldrich	Cat#D5796
Dulbecco’s Modified Eagle Medium, low glucose	Sigma-Aldrich	Cat#D6046
FreeStyle™ 293 Expression Medium	Gibco	Cat#12338018
L-Glutamine–Penicillin–Streptomycin solution	Sigma-Aldrich	Cat#G1146
GlutaMAX™ Supplement	Gibco	Cat#35050061
Opti-MEM™	Gibco	Cat#11058021
Fetal Bovine Serum	Gibco	Cat#12676029
Polyethylenimine, branched	Sigma-Aldrich	Cat#408727
Strep-Tactin®XT	IBA Lifesciences	Cat#2-1206-025
HEPES	Melford	Cat#34587-39108
Sodium Chloride	Honeywell	Cat#SZBF3340H
LB broth	Fisher Scientific UK	Cat#51577-51656
Mem Neaa (100X)	Gibco	Cat#2203945
Trypsin-EDTA	Gibco	Cat#2259288
TrypLE™ Express Enzyme	Gibco	Cat#12604013
L-Glutamine 200 mM (100X)	Gibco	Cat#2036885
SYPROorange (5000X in DMSO)	Thermo	Cat#S6651
Isopropyl β-d-1-thiogalactopyranoside	Meridian Bioscience	Cat#BIO-37036
Kanamycin	Melford	Cat#K22000
Lysozyme	Sigma-Aldrich	Cat#L6876
Tris-base	Melford	Cat#T60040
Imidazole	Sigma-Aldrich	Cat#56750
Triton-X-100	Sigma-Aldrich	Cat#8787
Turbonuclease	Sigma-Aldrich	Cat#T4330
RNAse A	Qiagen	Cat#158922
NaCl	Sigma-Aldrich	Cat#S9888
MgSO4	Sigma-Aldrich	Cat#746452
Na2HPO4	Melford	Cat#S23100
NaH2PO4	Melford	Cat#S23185
HBS-EP+ Buffer 10×	Cytiva	Cat# BR100669
Regeneration Solution (glycine-HCl pH 1.7)	Cytiva	Cat# BR100838
Sensor Chip Protein A	Cytiva	Cat#29127555
Biotin CAPture Kit, Series S	Cytiva	CAT#28920234
His-tagged SARS-CoV-2 BA.1 variant RBD	This paper	N/A
His-tagged SARS-CoV-2 BA.2 variant RBD	This paper	N/A
SARS-CoV-2 BA.1 variant Spike	This paper	N/A
SARS-CoV-2 BA.2 variant Spike	This paper	N/A
Streptavidin-APC	Biolegend	Cat# 405207
Streptavidin-APC	Biolegend	Cat# 405207
RNase inhibitor	Promega	Cat# N2611
Protein G Plus/Protein A Agarose	Millipore	Cat#IP10
Pierce™ Fab Preparation Kit	Thermo Fisher	Cat#44985
Twin-Strep-tag® Capture Kit	IBA-Lifesciences	Cat# 2-4370-000
PEGRx 2	Hampton Research	HR2-084
ProPlex™ HT-96	Molecular Dimensions	MD1-42
JCSG-plus™ HT-96	Molecular Dimensions	MD1-40

**Critical commercial assays**		

Bright-Glo Luciferase Assay System	Promega	Cat# E2620
HIV Type 1 p24 Antigen ELISA 2.0	ZeptoMetrix	Cat# 0801002

**Deposited data**		

Crystal structure of SARS-CoV-2 BA.4-RBD/Beta-27 Fab/Nanobody C1 complex	This paper	PDB: 7ZXU

**Experimental models: Cell lines**		

HEK293S GnTI- cells	ATCC	Cat#CRL-3022
HEK293 cells	ATCC	Cat#CRL-3216
Expi293F™ Cells	Gibco,	Cat#A14527
HEK293T/17 cells	ATCC	Cat#CRL-11268™
HEK293T cells	ATCC	Cat#CRL-11268
Hamster: ExpiCHO cells	Thermo Fisher	Cat#A29133

**Recombinant DNA**		

Vector: pHLsec	Aricescu et al., 2006	N/A
Vector: pNEO	Aricescu et al., 2006	N/A
Vector: pHLsec-SARS-CoV-2 spike of BA.1	This paper	N/A
Vector: pTTGneO-SARS-CoV-2 spike of BA.2	This paper	N/A
Vector: pTTGneO-SARS-CoV-2 RBD of BA.2	This paper	N/A
Vector: pNEO-SARS-CoV-2 RBD of BA.1	This paper	N/A
Vector: pCMV-VSV-G	Stewart et al., 2003	Addgene plasmid # 8454
pHR-SIN-ACE2	Alain Townsend	N/A
Vector: pOPING-ET	Nettleship et al., 2008	N/A
Vector: pJYDC1	Adgene	ID: 162458
Vector: p8.91	di Genova et al., 2020	Nigel Temperton
Vector: pCSFLW	di Genova et al., 2020	Nigel Temperton
Vector: pcDNA-SARS-CoV-2 spike of Wuhan strain	di Genova et al., 2020	Nigel Temperton
Vector: pcDNA-SARS-CoV-2 spike of Victoria strain (S247R)	Liu et al., 2021a	N/A
Vector: pcDNA-SARS-CoV-2 spike of Alpha strain (Δ69-70/144, N501Y, A570D, D614G, P681H, T716I, S982A, D1118H	Nutalai et al., 2022	N/A
Vector: pcDNA-SARS-CoV-2 spike of Beta strain (L18F, D80A, D215G, Δ242-244, R246I, K417N,E484K, N501Y, D614G, A701V)	Nutalai et al., 2022	N/A
Vector: pcDNA-SARS-CoV-2 spike of Gamma strain (L18F, T20N, P26S, D138Y, R190S, K417T, E484K, N501Y, D614G, H655Y, T1027I, V1176F)	Nutalai et al., 2022	N/A
Vector: pcDNA-SARS-CoV-2 spike of Delta+A222V strain (T19R, G142D, Del156-157/R158G, A222V, L452R, T478K, D614G, P681R, D950N)	Liu et al., 2021a	N/A
Vector: pcDNA-SARS-CoV-2 spike of BA.1 strain (A67V, Δ69-70, T95I, G142D/Δ143-145, Δ211/L212I, ins214EPE, G339D, S371L, S373P, S375F, K417N, N440K,G446S, S477N, T478K, E484A, Q493R, G496S, Q498R, N501Y, Y505H, T547K, D614G, H655Y, N679K, P681H, N764K, D796Y, N856K, Q954H, N969K, L981F)	Nutalai et al., 2022	N/A
Vector: pcDNA-SARS-CoV-2 spike of BA.1.1 strain (A67V, Δ69-70, T95I, G142D/Δ143-145, Δ211/L212I, ins214EPE, G339D, R346K, S371L, S373P, S375F, K417N, N440K,G446S, S477N, T478K, E484A, Q493R, G496S, Q498R, N501Y, Y505H, T547K, D614G, H655Y, N679K, P681H, N764K, D796Y, N856K, Q954H, N969K, L981F)	Nutalai et al., 2022	N/A
Vector: pcDNA-SARS-CoV-2 spike of BA.2 strain (T19I, Δ24-26, A27S, G142D, V213G, G339D, S371F, S373P, S375F, T376A, D405N, R408S, K417N, N440K, S477N, T478K, E484A, Q493R, Q498R, N501Y, Y505H, D614G, H655Y, N679K, P681H, N764K, D796Y, Q954H, N969K)	Nutalai et al., 2022	N/A
Vector: pcDNA-SARS-CoV-2 spike of BA.3 strain (A67V, Δ69-70, T95I, G142D/Δ143-145, Δ211/L212I, G339D, S371F, S373P, S375F, D405N, K417N, N440K, G446S, S477N, T478K, E484A, Q493R, Q498R, N501Y, Y505H, D614G, H655Y, N679K, P681H, N764K, D796Y, Q954H, N969K)	This paper	N/A
Vector: pcDNA-SARS-CoV-2 spike of BA.4/5 strain (T19I, Δ24-26, A27S, Δ69-70, G142D, V213G, G339D, S371F, S373P, S375F, T376A, D405N, R408S, K417N, N440K, L452R, S477N, T478K, E484A, F486V, Q498R, N501Y, Y505H, D614G, H655Y, N679K, P681H, N764K, D796Y, Q954H, N969K)	This paper	N/A
TM149 BirA pDisplay	University of Oxford, NDM (C. Siebold)	N/A

**Software and algorithms**		

COOT	Emsley et al., 2010	https://www2.mrc-lmb.cam.ac.uk/personal/pemsley/coot/
Xia2-dials	Winter et al., 2018	https://xia2.github.io
PHENIX	Liebschner et al., 2019	https://www.phenix-online.org/
PyMOL	Warren DeLano and Sarina Bromberg	https://pymol.org/
Data Acquisition Software 11.1.0.11	Fortebio	https://www.fortebio.com/products/octet-systems-software
Data Analysis Software HT 11.1.0.25	Fortebio	https://www.fortebio.com/products/octet-systems-software
Prism 9.0	GraphPad	https://www.graphpad.com/scientific-software/prism/
IBM SPSS Software 27	IBM	https://www.ibm.com
mabscape	This paper	https://snapcraft.io/mabscape
Biacore T200 Evaluation Software 3.1	Cytiva	www.cytivalifesciences.com
Flowjo 10.7.1	BD	https://www.flowjo.com
SnapGene software 5.3.2	Insightful Science	www.snapgene.com

**Other**		

X-ray data were collected at beamline I03, Diamond Light Source, under proposal lb27009 for COVID-19 rapid access	This paper	https://www.diamond.ac.uk/covid-19/for-scientists/rapid-access.html
TALON® Superflow Metal Affinity Resin	Clontech	Cat#635668
HiLoad® 16/600 Superdex® 200 pg	Cytiva	Cat#28-9893-35
Superdex 200 increase 10/300 GL column	Cytiva	Cat#28990944
HisTrap nickel HP 5-ml column	Cytiva	Cat#17524802
HiTrap Heparin HT 5-ml column	Cytiva	Cat#17040703
Amine Reactive Second-Generation (AR2G) Biosensors	Fortebio	Cat#18-5092
Octet RED96e	Fortebio	https://www.fortebio.com/products/label-free-bli-detection/8-channel-octet-systems
Buffer exchange system “QuixStand”	GE Healthcare	Cat#56-4107-78
Cartesian dispensing system	Genomic solutions	Cat#MIC4000
Hydra-96	Robbins Scientific	Cat#Hydra-96
96-well crystallization plate	Greiner bio-one	Cat#E20113NN
Crystallization Imaging System	Formulatrix	Cat#RI-1000
Sonics vibra-cell vcx500 sonicator	VWR	Cat#432-0137
Biacore T200	Cytiva	https://www.cytivalifesciences.com/en/us/shop/protein-analysis/spr-label-free-analysis/systems/biacore-t200-p-05644
QuixStand	GE Healthcare	Cat# 56-4107-78

### Resource availability

#### Lead contact

Resources, reagents and further information requirement should be forwarded to and will be responded by the lead contact, David I. Stuart (dave@strubi.ox.ac.uk).

#### Materials availability

Reagents generated in this study are available from the lead contact with a completed Materials Transfer Agreement.

### Experimental model and subject details

#### Bacterial strains and cell culture

Vero (ATCC CCL-81) and VeroE6/TMPRSS2 cells were cultured at 37 °C in Dulbecco’s Modified Eagle medium (DMEM) high glucose (Sigma-Aldrich) supplemented with 10% fetal bovine serum (FBS), 2 mM GlutaMAX (Gibco, 35050061) and 100 U/ml of penicillin–streptomycin. Human mAbs were expressed in HEK293T cells cultured in UltraDOMA PF Protein-free Medium (Cat# 12-727F, LONZA) at 37 °C with 5% CO_2_. HEK293T (ATCC CRL-11268) cells were cultured in DMEM high glucose (Sigma-Aldrich) supplemented with 10% FBS, 1% 100X Mem Neaa (Gibco) and 1% 100X L-Glutamine (Gibco) at 37 °C with 5% CO_2_. To express RBD, RBD variants and ACE2, HEK293T cells were cultured in DMEM high glucose (Sigma) supplemented with 2% FBS, 1% 100X Mem Neaa and 1% 100X L-Glutamine at 37 °C for transfection. Omicron RBD and human mAbs were also expressed in HEK293T (ATCC CRL-11268) cells cultured in FreeStyle 293 Expression Medium (ThermoFisher, 12338018) at 37 °C with 5% CO_2_. *E.coli DH5α* bacteria were used for transformation and large-scale preparation of plasmids. A single colony was picked and cultured in LB broth at 37 °C at 200 rpm in a shaker overnight.

#### Plasma from early-pandemic and Alpha cases

Participants from the first wave of SARS-CoV2 in the U.K. and those sequence confirmed with B.1.1.7 lineage in December 2020 and February 2021 were recruited through three studies: Sepsis Immunomics [Oxford REC C, reference:19/SC/0296]), ISARIC/WHO Clinical Characterisation Protocol for Severe Emerging Infections [Oxford REC C, reference 13/SC/0149] and the Gastro-intestinal illness in Oxford: COVID sub study [Sheffield REC, reference: 16/YH/0247]. Diagnosis was confirmed through reporting of symptoms consistent with COVID-19 and a test positive for SARS-CoV-2 using reverse transcriptase polymerase chain reaction (RT-PCR) from an upper respiratory tract (nose/throat) swab tested in accredited laboratories. A blood sample was taken following consent at least 14 days after symptom onset. Clinical information including severity of disease (mild, severe or critical infection according to recommendations from the World Health Organisation) and times between symptom onset and sampling and age of participant was captured for all individuals at the time of sampling. Following heat inactivation of plasma/serum samples they were aliquoted so that no more than 3 freeze thaw cycles were performed for data generation.

#### Sera from Beta-, Gamma-, and Delta- and BA.1-infected cases

Beta and Delta samples from UK infected cases were collected under the “Innate and adaptive immunity against SARS-CoV-2 in healthcare worker family and household members” protocol affiliated to the Gastro-intestinal illness in Oxford: COVID sub study discussed above and approved by the University of Oxford Central University Research Ethics Committee. All individuals had sequence confirmed Beta/Delta infection or PCR-confirmed symptomatic disease occurring whilst in isolation and in direct contact with Beta/Delta sequence-confirmed cases. Additional Beta infected serum (sequence confirmed) was obtained from South Africa. At the time of swab collection patients signed an informed consent to consent for the collection of data and serial blood samples. The study was approved by the Human Research Ethics Committee of the University of the Witwatersrand (reference number 200313) and conducted in accordance with Good Clinical Practice guidelines. Gamma samples were provided by the International Reference Laboratory for Coronavirus at FIOCRUZ (WHO) as part of the national surveillance for coronavirus and had the approval of the FIOCRUZ ethical committee (CEP 4.128.241) to continuously receive and analyse samples of COVID-19 suspected cases for virological surveillance. Clinical samples were shared with Oxford University, UK under the MTA IOC FIOCRUZ 21-02.

#### Sera from BA.1-infected cases, study subjects

Following informed consent, individuals with omicron BA.1 were co-enrolled into the ISARIC/WHO Clinical Characterisation Protocol for Severe Emerging Infections [Oxford REC C, reference 13/SC/0149] and the “Innate and adaptive immunity against SARS-CoV-2 in healthcare worker family and household members” protocol affiliated to the Gastro-intestinal illness in Oxford: COVID sub study [Sheffield REC, reference: 16/YH/0247] further approved by the University of Oxford Central University Research Ethics Committee. Diagnosis was confirmed through reporting of symptoms consistent with COVID-19 or a positive contact of a known Omicron case, and a test positive for SARS-CoV-2 using reverse transcriptase polymerase chain reaction (RT-PCR) from an upper respiratory tract (nose/throat) swab tested in accredited laboratories and lineage sequence confirmed through national reference laboratories. A blood sample was taken following consent at least 10 days after PCR test confirmation. Clinical information including severity of disease (mild, severe or critical infection according to recommendations from the World Health Organisation) and times between symptom onset and sampling and age of participant was captured for all individuals at the time of sampling.

#### Sera from Pfizer vaccinees

Pfizer vaccine serum was obtained from volunteers who had received three doses of the BNT162b2 vaccine. Vaccinees were Health Care Workers, based at Oxford University Hospitals NHS Foundation Trust, not known to have prior infection with SARS-CoV-2 and were enrolled in the OPTIC Study as part of the Oxford Translational Gastrointestinal Unit GI Biobank Study 16/YH/0247 [research ethics committee (REC) at Yorkshire & The Humber – Sheffield] which has been amended for this purpose on 8 June 2020. The study was conducted according to the principles of the Declaration of Helsinki (2008) and the International Conference on Harmonization (ICH) Good Clinical Practice (GCP) guidelines. Written informed consent was obtained for all participants enrolled in the study. Participants were sampled approximately 28 days (range 25-56) after receiving a third “booster dose of BNT162B2 vaccine. The mean age of vaccinees was 37 years (range 22-66), 21 male and 35 female.

#### AstraZeneca-Oxford vaccine study procedures and sample processing

Full details of the randomized controlled trial of ChAdOx1 nCoV-19 (AZD1222), were previously published (PMID: 33220855/PMID: 32702298). These studies were registered at ISRCTN (15281137 and 89951424) and ClinicalTrials.gov (NCT04324606 and NCT04400838). Written informed consent was obtained from all participants, and the trial is being done in accordance with the principles of the Declaration of Helsinki and Good Clinical Practice. The studies were sponsored by the University of Oxford (Oxford, UK) and approval obtained from a national ethics committee (South Central Berkshire Research Ethics Committee, reference 20/SC/0145 and 20/SC/0179) and a regulatory agency in the United Kingdom (the Medicines and Healthcare Products Regulatory Agency). An independent DSMB reviewed all interim safety reports. A copy of the protocols was included in previous publications (Folegatti et al., 2020). Data from vaccinated volunteers who received three vaccinations are included in this study. Blood samples were collected and serum separated approximately 28 days (range 26-34 days) following the third dose.

### Method details

#### Plasmid construction and pseudotyped lentiviral particles production

Pseudotyped lentivirus expressing SARS-CoV-2 S proteins from ancestral strain (Victoria, S247R), BA.1, BA.1.1, and BA.2 were constructed as described previously (Nie et al., 2020; Liu et al., 2021b; Nutalai et al., 2022), with some modifications. A similar strategy was applied for BA.3 and BA.4/5, briefly, BA.3 mutations were constructed using the combination fragments from BA.1 and BA.2. The resulting mutations are as follows, A67V, Δ69-70, T95I, G142D, Δ143-145, Δ211/L212I, G339D, S371F, S373P, S375F, D405N, K417N, N440K, G446S, S477N, T478K, E484A, Q493R, Q498R, N501Y, Y505H, D614G, H655Y, N679K, P681H, N764K, D796Y, Q954H, and N969K. Although BA.4/5 S protein shared some amino acid mutations with BA.2 (Nutalai et al., 2022), to generate BA.4/5 we added mutations Δ69-70, L452R, F486V, and R498Q. The resulting S gene-carrying pcDNA3.1 was used for generating pseudoviral particles together with the lentiviral packaging vector and transfer vector encoding luciferase reporter. Integrity of constructs was sequence confirmed.

#### Pseudoviral neutralization test

The details of the pseudoviral neutralization test are as described previously (Liu et al., 2021b) with some modifications. Briefly, the neutralizing activity of potent monoclonal antibodies generated from donors who had recovered from Omicron were assayed against Victoria, Omicron-BA.1, BA.1.1, BA.2, BA.3 and BA.4/5. Four-fold serial dilutions of each mAb were incubated with pseudoviral particles at 37°C, 5% CO2 for 1 hr. Stable HEK293T/17 cells expressing human ACE2 were then added to the mixture at 1.5 x 10^4^ cells/well. 48 hr post transduction, culture supernatants were removed and 50 μL of 1:2 Bright-GloTM Luciferase assay system (Promega, USA) in 1x PBS was added to each well. The reaction was incubated at room temperature for 5 mins and firefly luciferase activity was measured using CLARIOstar® (BMG Labtech, Ortenberg, Germany). The percentage neutralization was calculated relative to the control. Probit analysis was used to estimate the dilution that inhibited half maximum pseudotyped lentivirus infection (PVNT50).

To determine the neutralizing activity of convalescent plasma/serum samples or vaccine sera, 3-fold serial dilutions of samples were incubated with pseudoviral particles for 1 hr and the same strategy as mAb was applied.

#### Cloning of RBDs

To generate His-tagged constructs of BA.4/5 RBD, site-directed PCR mutagenesis was performed using the BA.2 RBD construct as the template (Nutalai et al., 2022), with the introduction of L452R, F486V and R493Q mutations. The gene fragment was amplified with pNeoRBD333Omi_F (5’- GGTTGCGTAGCTGAAACCGGTCATCACCATCACCATCACACCAATCTGTGCCCTTTCGAC-3’) and pNeoRBD333_R (5’-GTGATGGTGGTGCTTGGTACCTTATTACTTCTTGCCGCACACGGTAGC-3’), and cloned into the pNeo vector (Supasa et al., 2021). To generate the BA.4/5 RBD construct containing a BAP-His tag, the gene fragment was amplified with RBD333_F (5’-GCGTAGCTGAAACCGGCACCAATCTGTGCCCTTTCGAC-3’) and RBD333_BAP_R (5’- GTCATTCAGCAAGCTCTTCTTGCCGCACACGGTAGC-3’), and cloned into the pOPINTTGneo-BAP vector (Huo et al., 2020a). Cloning was performed using the ClonExpress II One Step Cloning Kit (Vazyme). The Constructs were verified by Sanger sequencing after plasmid isolation using QIAGEN Miniprep kit (QIAGEN).

#### Production of RBDs

Plasmids encoding RBDs were transfected into Expi293F™ Cells (ThermoFisher) by PEI, cultured in FreeStyle™ 293 Expression Medium (ThermoFisher) at 30 °C with 8% CO_2_ for 4 days. To express biotinylated RBDs, the RBD-BAP plasmid was co-transfected with pDisplay-BirA-ER (Addgene plasmid 20856; coding for an ER-localized biotin ligase), in the presence of 0.8 mM D-biotin (Sigma-Aldrich). The conditioned medium was diluted 1:2 into binding buffer (50 mM sodium phosphate, 500 mM sodium chloride, pH 8.0). RBDs were purified with a 5 mL HisTrap nickel column (GE Healthcare) through His-tag binding, followed by a Superdex 75 10/300 GL gel filtration column (GE Healthcare) in 10 mM HEPES and 150 mM sodium chloride.

#### Surface plasmon resonance

The surface plasmon resonance experiments were performed using a Biacore T200 (GE Healthcare). All assays were performed with running buffer of HBS-EP (Cytiva) at 25 °C.

To determine the binding kinetics between the RBDs and mAb Omi-32 / Omi-42, a Biotin CAPture Kit (Cytiva) was used. Biotinylated RBD was immobilised onto the sample flow cell of the sensor chip. The reference flow cell was left blank. The mAb Fab was injected over the two flow cells at a range of five concentrations prepared by serial two-fold dilutions, at a flow rate of 30 μl min^−1^ using a single-cycle kinetics programme. Running buffer was also injected using the same programme for background subtraction. All data were fitted to a 1:1 binding model using Biacore T200 Evaluation Software 3.1.

To determine the binding kinetics between RBDs and ACE2 / other mAbs, a Protein A sensor chip (Cytiva) was used. ACE2-Fc or mAb in the IgG form was immobilised onto the sample flow cell of the sensor chip. The reference flow cell was left blank. RBD was injected over the two flow cells at a range of five concentrations prepared by serial two-fold dilutions, at a flow rate of 30 μl min^−1^ using a single-cycle kinetics programme. Running buffer was also injected using the same programme for background subtraction. All data were fitted to a 1:1 binding model using Biacore T200 Evaluation Software 3.1.

To determine the binding affinity of BA.4/5 RBD and mAb Omi-12, a Protein A sensor chip (Cytiva) was used. The Ig Omi-12 was immobilised onto the sample flow cell of the sensor chip. The reference flow cell was left blank. RBD was injected over the two flow cells at a range of seven concentrations prepared by serial twofold dilutions, at a flow rate of 30 μl min^−1^. Running buffer was also injected using the same programme for background subtraction. All data were fitted to a 1:1 binding model using Prism9 (GraphPad).

To compare the binding profiles between BA.2 and BA.4/5 RBD for mAb Omi-06 / Omi-25 / Omi-26, a Protein A sensor chip (Cytiva) was used. mAb in the IgG form was immobilised onto the sample flow cell of the sensor chip to a similar level (∼350 RU). The reference flow cell was left blank. A single injection of RBD was performed over the two flow cells at 200 nM, at a flow rate of 30 μl min^−1^. Running buffer was also injected using the same programme for background subtraction. The sensorgrams were plotted using Prism9 (GraphPad).

To compare the binding profiles between BA.2 and BA.4/5 RBD for mAb Omi-02 / Omi-23 / Omi-31, a Biotin CAPture Kit (Cytiva) was used. Biotinylated BA.2 and BA.4/5 RBD was immobilised onto the sample flow cell of the sensor chip to a similar level (∼120 RU). The reference flow cell was left blank. A single injection of mAb Fab was performed over the two flow cells at 200 nM, at a flow rate of 30 μl min^−1^. Running buffer was also injected using the same programme for background subtraction. The sensorgrams were plotted using Prism9 (GraphPad).

#### IgG mAbs and Fabs production

AstraZeneca and Regeneron antibodies were provided by AstraZeneca, Vir, Lilly and Adagio antibodies were provided by Adagio. For the in-house antibodies, heavy and light chains of the indicated antibodies were transiently transfected into 293Y cells and antibody purified from supernatant on protein A as previously described (Nutalai et al., 2022). Fabs were digested from purified IgGs with papain using a Pierce Fab Preparation Kit (Thermo Fisher), following the manufacturer’s protocol.

#### Crystallization, X-ray data collection, and structure determination

Crystals of BA.4 RBD/Beta-27 complex were grown from 4% (v/v) 2-propanol, 0.1M BIS-Tris propane, pH9.0, 20% (w/v) PEG monomethyl ether 5000 using the sitting drop method and nanobody NbC1 as a crystallisation chaperon. Diffraction data were collected at 100 K at beamline I03 of Diamond Light Source, UK, using the automated queue system that allows unattended automated data collection (https://www.diamond.ac.uk/Instruments/Mx/I03/I03-Manual/Unattended-Data-Collections.html). Structures were determined by molecular replacement with PHASER (McCoy et al., 2007). VhVl and ChCl domains of Beta-27 (Liu et al., 2021a) and RBD/NbC1 complex (PDB, 7OAP) were used as search models. Model rebuilding was done with COOT (Emsley et al., 2010) and refinement with Phenix (Liebschner et al., 2019).

Data collection and structure refinement statistics are given in Table S1 and structural details in Figure S1. Structural comparisons used SHP (Stuart et al., 1979) and figures were prepared with PyMOL (The PyMOL Molecular Graphics System, Version 1.2r3pre, Schrödinger, LLC).

#### Antigenic mapping

Antigenic mapping of Omicron was carried out through an extension of a previous algorithm (Liu et al., 2021a). In short, coronavirus variants were assigned three-dimensional coordinates whereby the distance between two points indicates the base drop in neutralization titre. Each serum was assigned a strength parameter which provided a scalar offset to the logarithm of the neutralization titre. These parameters were refined to match predicted neutralization titres to observed values by taking an average of superimposed positions from 30 separate runs. The three-dimensional positions of the variants of concern: Victoria, Alpha, Beta, Gamma, Delta and Omicron were plotted for display.

### Quantification and statistical analysis

Statistical analyses are reported in the results and figure legends. Neutralization was measured on pseudovirus. The percentage reduction was calculated and IC_50_ determined using the probit program from the SPSS package. The Wilcoxon matched-pairs signed rank test was used for the analysis and two-tailed P values were calculated on geometric mean values.

## Consortia

The full contributor list for the ISARIC4C Consortium is available at https://isaric4c.net/about/authors/. The members of the OPTIC Consortium are Christopher Conlon, Alexandra Deeks, John Frater, Lisa Frending, Siobhan Gardiner, Anni Jämsén, Katie Jeffery, Tom Malone, Eloise Phillips, Lucy Rothwell, and Lizzie Stafford.

## Data Availability

The coordinates and structure factors are available from the PDB with accession code 7ZXU. Mabscape is available from https://github.com/helenginn/mabscape, https://snapcraft.io/mabscape. The data that support the findings of this study are available from the corresponding authors on request.
