# Supplementary material for: Antibody escape of SARS-CoV-2 Omicron BA.4 and BA.5 from vaccine and BA.1 serum
Source: Cell. 2022 Jul 7;185(14):2422–2433.e13. doi: 10.1016/j.cell.2022.06.005 (PMC9181312; doi:10.1016/j.cell.2022.06.005)
Supplement: Document S1. Table S1 [file mmc1.pdf]

Cell, Volume *185*

Supplemental information

Antibody escape of SARS-CoV-2 Omicron BA.4 and BA.5 from vaccine and BA.1 serum

Aekkachai Tuekprakhon, Rungtiwa Nutalai, Aiste Dijokaite-Guraliuc, Daming Zhou, Helen M. Ginn, Muneeswaran Selvaraj, Chang Liu, Alexander J. Mentzer, Piyada Supasa, Helen M.E. Duyvesteyn, Raksha Das, Donal Skelly, Thomas G. Ritter, Ali Amini, Sagida Bibi, Sandra Adele, Sile Ann Johnson, Bede Constantinides, Hermione Webster, Nigel Temperton, Paul Klenerman, Eleanor Barnes, Susanna J. Dunachie, Derrick Crook, Andrew J. Pollard, Teresa Lambe, Philip Goulder, Neil G. Paterson, Mark A. Williams, David R. Hall, OPTIC Consortium, ISARIC4C Consortium, Elizabeth E. Fry, Jiandong Huo, Juthathip Mongkolsapaya, Jingshan Ren, David I. Stuart, and Gavin R. Screaton

Table S1: Structure determination and refinement. Related to Methods.

**Structure**	**BA.4/5 RBD/Beta-27/NbC1, PDB: 7ZXU**
**Data collection**	
Space group	$P2_12_12_1$
Cell dimensions	
a, b, c (Å)	84.1, 100.4, 105.4
α, β, γ (°)	90, 90, 90
Resolution (Å)	66–1.89 (1.92–1.89)[a]
R_{merge}	0.313 (---)
R_{pim}	0.061 (0.848)
I/s(I)	7.7 (0.5)
$CC_{1/2}$	0.996 (0.418)
Completeness (%)	100 (99.4)
Redundancy	27.4 (28.2)
**Refinement**	
Resolution (Å)	66–1.89
No. reflections	68286/3756
R_{work} / R_{free}	0.183/0.210
No. atoms	
Protein	5805
Ligand/ion/water	672
B factors (Å^2)	
Protein	39
Ligand/ion/water	47
r.m.s. deviations	
Bond lengths (Å)	0.002
Bond angles (Å)	0.5
